# Gestational trophoblastic neoplasia with primary lung cancer and mesenchymal tumor of sigmoid colon: a case report and literature review

**DOI:** 10.1186/s12905-023-02204-7

**Published:** 2023-02-20

**Authors:** HongYe Li, Meng Sun, Jing Jiang, Bin Shi, BaoHua Wang, Lei Wang, WenXin Wu, WenYan Wang

**Affiliations:** 1 Department of Obstetics and Gynecology, The Second Hospital of Hebei Medical University, Shijiazhuang, Hebei China; 2 Department of Thoracic Surgery, The Second Hospital of Hebei Medical University, Shijiazhuang, Hebei China; 3 Department of General Surgery, The Second Hospital of Hebei Medical University, Shijiazhuang, Hebei China; 4 Department of Pathology, The Second Hospital of Hebei Medical University, Shijiazhuang, Hebei China; 5 Department of Medical Imaging, The Second Hospital of Hebei Medical University, Shijiazhuang, Hebei China

**Keywords:** Gestational trophoblastic neoplasia, Primary lung cancer, Mesenchymal tumor

## Abstract

**Background:**

Gestational trophoblastic neoplasia (GTN) is rare, and it is even rarer for GTN to merge with primary malignant tumors in other organs. Herein is described a rare clinical case of GTN combined with primary lung cancer and mesenchymal tumor of the sigmoid colon, followed with literature review.

**Case presentation:**

The patient was hospitalized due to diagnosis of GTN with primary lung cancer. Firstly, two cycles of chemotherapy including 5-fluorouracil (5-FU) and actinomycin-D(Act-D) was given. Laparoscopic total hysterectomy and right salpingo-oophorectomy was performed during the third chemotherapy. During the operation, a 3*2 cm nodule was removed which was protruded from the serous surface of the sigmoid colon, and the nodule was confirmed mesenchymal tumor pathologically, in accord with gastrointestinal stromal tumor. During the treatment of GTN, Icotinib tablets were taken orally to control the progression of lung cancer. After 2 cycles of consolidation chemotherapy of GTN, she received thoracoscopic lower lobe of right lung lobectomy and the mediastinum lymph nodes removal. She undertook gastroscopy and colonoscopy and the tubular adenoma of the descending colon was removed. At present, the regular follow-up is taken and she remains free of tumors.

**Conclusions:**

GTN combined with primary malignant tumors in other organs are extremely rare in clinical practice. When imaging examination reveals a mass in other organs, clinicians should be aware of the possibility of a second primary tumor. It will increase the difficulty of GTN staging and treatment. We emphasis the importance of the collaboration of multidisciplinary teams. Clinicians should choose a reasonable treatment plan according to the priorities of different tumors.

## Background

Gestational trophoblastic disease (GTD) is a group of diseases derived from placental trophoblasts. Histologically, it includes the premalignant partial hydatidiform mole (PHM) and complete hydatidiform mole (CHM), as well as the malignant invasive mole, choriocarcinoma, placental site trophoblastic tumor (PSTT), and epithelioid trophoblastic tumor (ETT). All malignant GTD are collectively known as gestational trophoblastic neoplasia (GTN). GTN is rare, and it is even rarer for GTN to merge with primary malignant tumors in other organs, only 6 cases have been published to date in the literature and two of which were reported in China. For GTN, timely and effective treatment will produce a relatively promising prognosis. However, GTN combined with primary malignant tumors in other organs will increase the difficulty of GTN staging and treatment, and bring more challenges to clinicians. Now the clinical data of one case of GTN with primary lung cancer and mesenchymal tumors of the sigmoid colon in a tertiary hospital in China are herein reported, followed with literature review to explore the incidence, diagnosis and treatment of GTN combined with primary malignant tumors in other organs. Due to the low incidence and lack of clinical experience, we believe that this report will be helpful to the diagnosis and treatment of similar cases in the future. Our case reminds clinicians that should be make comprehensive judgment and reduce misdiagnosis and missed diagnosis.

## Case presentation

A 47-year-old woman, gravida 3, para 1, gave birth 26 years ago. She was admitted to our hospital on March 06, 2019, mainly because the blood beta-human chorionic gonadotropin (β-hCG) rose 9 days after a curettage of hydatidiform mole which was performed 3 months ago. Ordinarily menstruation is regular and the last menstruation period was September 24, 2018. More than 3 months ago, the patient went to the local hospital for 63 days of menopause and 20 days of vaginal bleeding. B-ultrasound suspected hydatidiform mole, blood β-hCG was 272,800 U/L, pelvic Magnetic resonance imaging (MRI) revealed cystic and solid mass in the uterine cavity with invasion of the myometrium and considered erosion mole. On November 28, 2018, a sono-guided uterine suction was performed and the pathological examination showed hydatidiform mole. After the curettage, a prophylactic chemotherapy was given using actinomycin-D (Act-D) injection for 5 days and the blood β-hCG decreased gradually. Two days after the suction, the blood β-hCG was 133,531 U/L, 5 days was 45,778 U/L, and 7 days was 29,650 U/L. Half a month later, the ultrasonography of uterine suggested incomplete suction. So on December 18, 2018, the dilatation and curettage (D&C) was conducted again and the pathology was the same as before.

After that, β-hCG titers were obtained regularly. Unfortunately, β-hCG increased from 7341 U/L (February 25, 2019) to 11,333 U/L (March 05, 2019). So she visited our hospital on March 6, 2019, and the blood β-hCG was 41,611 U/L at that time. Transvaginal sonography showed intramural abnormal strong echo area in uterine fundus of 6.26*6.04 cm in size with abundant blood flow and arteriovenous fistula was formulated. Then she was hospitalized due to diagnosis of GTN. The patient had undergone left salpingo-oophorectomy because of benign adnexal mass 7 years before.

It is worth noting that the computed tomography (CT) scan of her thorax before the first D&C indicated a space-occupying lesion in the inferior lobe of right lung, though she had no fever, no cough, no sputum, no hemoptysis or any other symptoms. CT of the head and abdomen showed no abnormalities. The chest CT rechecked during the second D&C still showed the same lesion. Percutaneous lung biopsy was thus undertaken, and the pathological result showed: (the basal segment of the right lower lobe) well-differentiated adenocarcinoma, consistent with primary lung cancer. Immunohistochemical results were CK7 (+), TTF-1 (+), TG (−), WT-1 (−), P53 (weak positive, suggesting wild type) Pax-8 (1). On December 20, 2018, she went to the local tertiary hospital. The thoracic CT also showed the space-occupying lesion in the inferior lobe of the right lung considered malignancy, and multiple small nodules in both lungs considered metastasis. Both the cranial and abdominal CT and bone scan were negative. In order to confirm the diagnosis, a repeated percutaneous needle biopsy was done and the pathological result also showed primary lung adenocarcinoma. Therefore, she received chemotherapy of TP regimen, with Paclitaxel 210 mg (D1) and cisplatin 40 mg (D2-D4) for twice. The third time chemotherapy could not be performed because of myelosuppression. She began to take targeted agent icotinib tablets (125 mg tid) from February 19, 2019, since the genetic testing of the lung biopsy specimen suggested EGFR mutation.

**Fig. 1:**
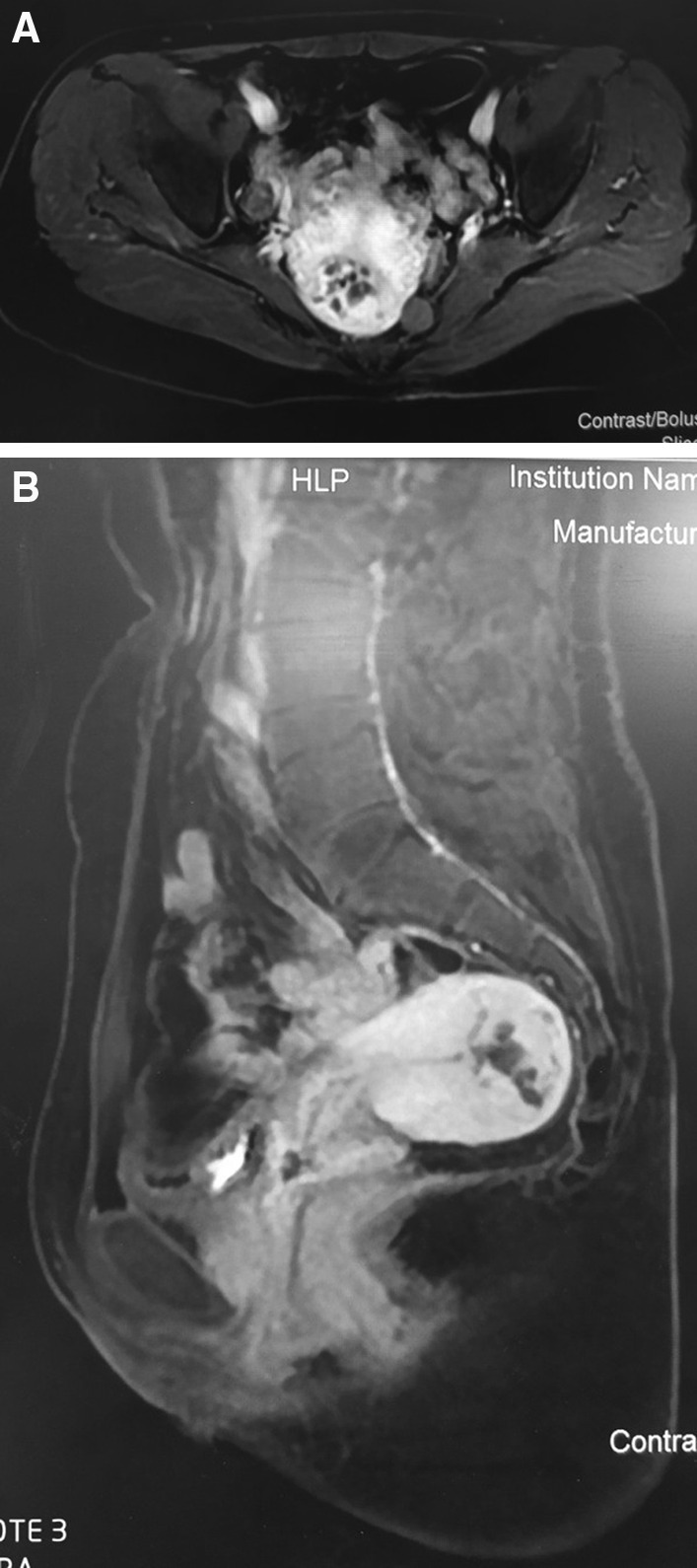
The pelvic MRI showed uterine lesion considered GTN, and abnormally increased blood vessels were detected in that lesion (**A**: MRI scan in axial view, **B**: MRI scan in sagittal view)

**Fig. 2:**
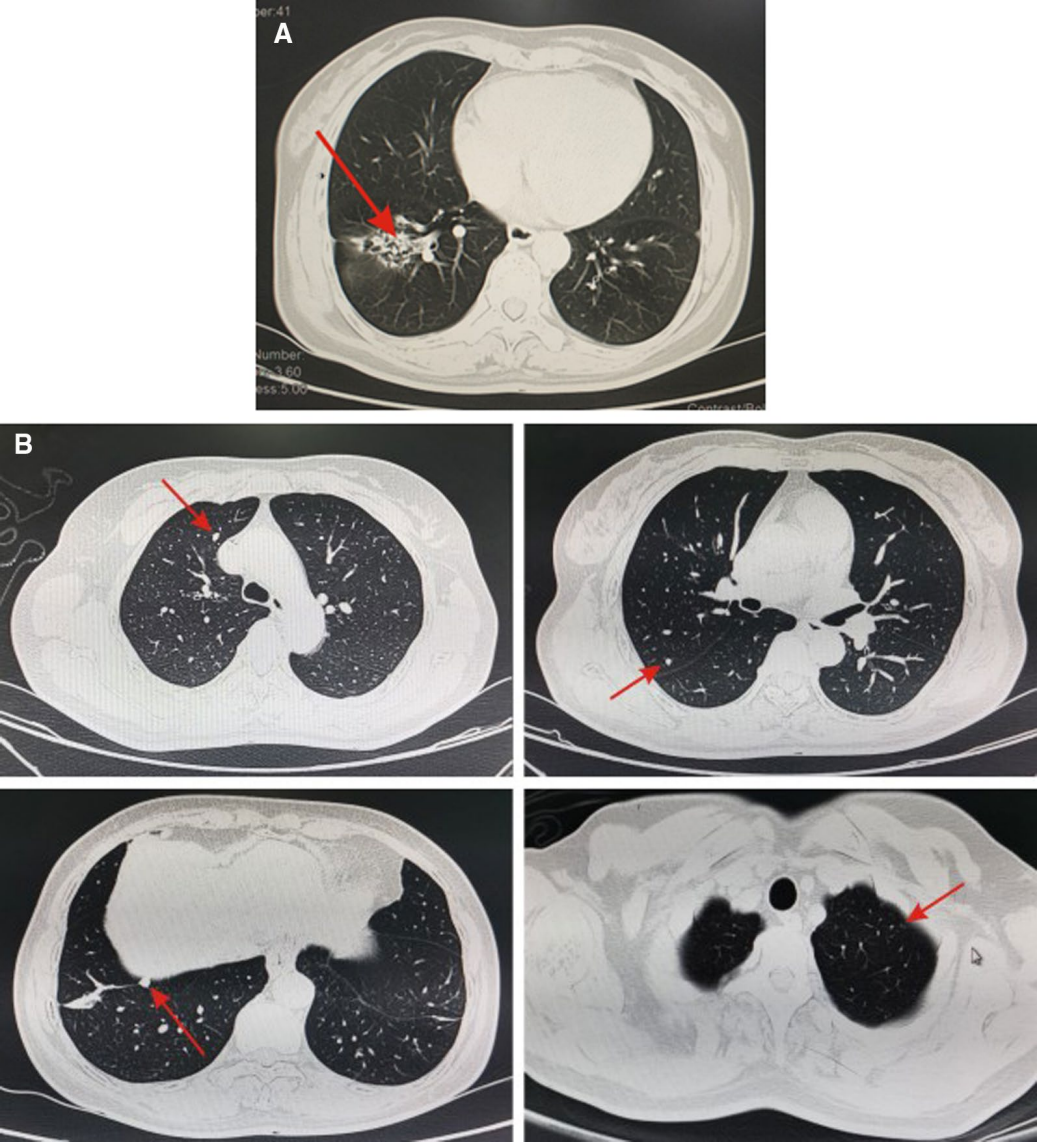
The chest CT scan showed the space-occupying lesion in the inferior lobe of the right lung (arrows) (**A**) and multiple small nodules in both lungs (arrows) (**B**)

In our hospital, the patient reexamined the pelvic MRI on March 11, 2019, which showed uterine lesion considered GTN, and abnormally increased blood vessels were detected in that lesion (Fig. 1). The chest CT images of lung in our hospital were showed in Fig. 2. Pathological section of both D&C and lung biopsy were read again by two experienced pathologists. The morphology of D&C tissue conformed to hydatidiform mole, and trophoblast cells and edema villus could be seen in degenerative tissues. Puncture biopsy conformed to lung adenocarcinoma with papillary structures in part.

Based on: ① 47-year-old woman (1 point), ② a D&C history with pathologic result of hydatidiform mole (0 point), ③ a β-hCG level of 41,611 U/L (2 points), ④ transvaginal ultrasound and pelvic MRI both suggested an intramural lesion of more than 6 cm in uterine fundus (2 points), ⑤ multiple small nodules in both lungs in thoracic CT but none in chest X-ray (0 point), ⑥ the space-occupying lesion in the inferior lobe of the right lung and the biopsy pathology showed primary lung adenocarcinoma. The preliminary diagnosis were GTN (stage III: 5 points) and lung cancer.

**Fig. 3:**
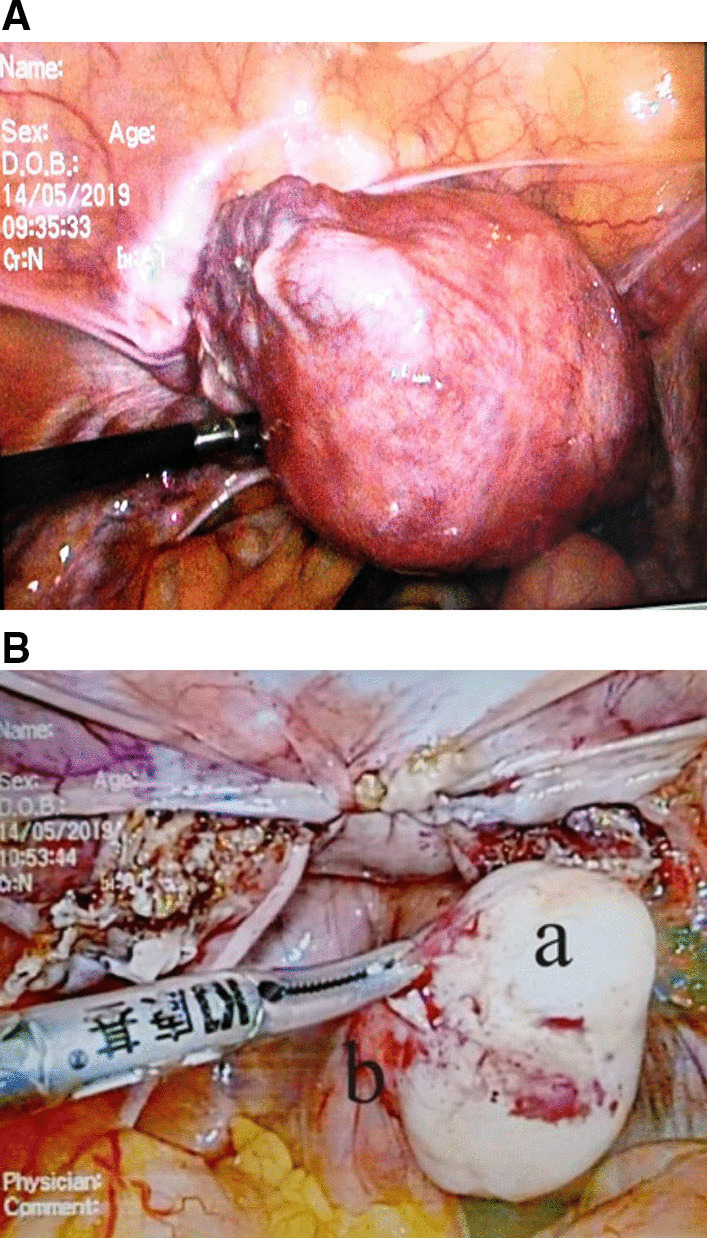
Intraoperative laparoscopic picture showed: **A** the uterus was slightly larger, with abundant blood vessels on its surface. **B** A hard and white-colored nodule (**a**) originated from the sigmoid colon (**b**)

**Fig. 4:**
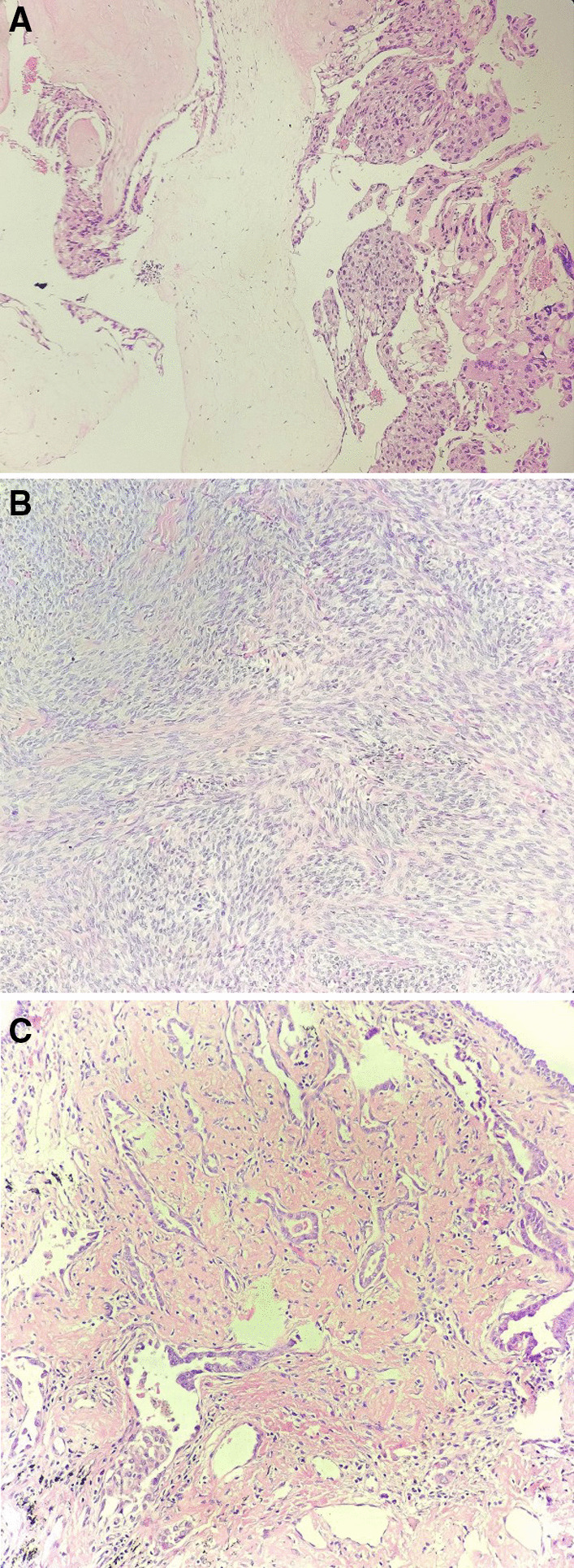
Hematoxylin eosin staining showed (magnification × 10): **A** Trophoblastic cells with dysplasia and the hydropic villi. **B** The tumor cells originated from the sigmoid colon were spindle-shaped and arranged in bundles. **C** Infiltrative atypical glands in the right lung showed an acinar and adherent growth pattern

Consider the high level of hCG, firstly, two cycles of chemotherapy including 5-fluorouracil (5-FU) and Act-D was given. Before the third cycle of chemotherapy, the blood β-hCG (May 07, 2019) dropped to 694.02 U/L. The patient was 47 years old and had no reproductive desire. In order to reduce the tumor burden, shorten the course and reduce the side effects of chemotherapy, the patient agreed to remove the uterus, and ask for right salpingo-oophorectomy resection concurrently. Laparoscopic total hysterectomy and right salpingo-oophorectomy was performed on May 14, 2019. During the operation, we found the uterus was slightly larger, with abundant blood vessels in its surface (Fig. 3A), the left adnexa was absent, and the right adnexa was normal in appearance. Moreover, a 3*2 cm nodule was noticed, which was hard and had a complete and smooth capsule, protruded from the serous surface of the sigmoid colon (Fig. 3B). We called for a general surgeon to remove the nodule. The operation went smoothly and the intraoperative hemorrhage was 50 ml. Postoperative pathological results showed: ① infiltration of hyperplastic trophoblast cells in the fundus endometrium and muscles, accompanied by necrosis, occasional karyokinesis, and conformed to the changes of GTN after chemotherapy (Fig. 4A). Immunohistochemical results were CKpan (+), HCG (+), Inhibin-a (+), Ki-67 (hot zone + 70%), P63 (very few cells+), PLAP (−); ② the proliferative phase endometrium, uterine myometrial leiomyoma, chronic cervicitis with Nabothian cyst; ③ chronic inflammation of the right fallopian tube, and no abnormal findings on the right ovary; ④ the nodule (2.3 × 2 × 1.5 cm) on the surface of sigmoid colon was mesenchymal tumor, in accord with gastrointestinal stromal tumor (Fig. 4B), immunohistochemically CD117 (−), CD34 (+), Desmin (−), DOG1 (−), Ki-67 (+ 15%), S-100 (−), SMA (−), SMMS-1 (−), CKpan (−). Chemotherapy was continued on the 3rd day after surgery. Before the 4th chemotherapy, the blood β-hCG dropped to normal, and then she was given 2 cycles of consolidation chemotherapy, and the last chemotherapy course was finished on July 13, 2019. There was no abnormality in the monthly gynecological B-ultrasound, and the serum hCG titer was in the normal range.

During the treatment of GTN, Icotinib tablets were taken orally to control the progression of lung cancer, and the patient regularly recheck the chest CT, the CT showed that the right lung lower lobe shadow slightly reduced, the density slightly lower and the multiple nodules in both lungs gradually disappeared. Three months later, on August 12, 2019, thoracoscopic lower lobe of right lung lobectomy and the mediastinum lymph nodes removal were performed by thoracic doctors. Pathology results revealed the lower lobe adenocarcinoma of right lung (2.5 × 2.0 × 1.0 cm, nipple type 60%, adherent type 30%, acinus type 10%) (Fig. 4C), no definite cancer embolus in the vessels, and the bronchial stump, pleura, along with the 2, 4, 7, 8, 9, 11, 12 groups of lymph nodes did not show malignancy. Immunohistochemical results showed CA-125 (−), CK20 (−), CK7 (−), CKpan (−), HCG (−), Ki-67 (about 3%+), NapsinA (+), P63 (−), TTF-1 (−), (AFP-). Elastic fiber staining showed pleural aggression (−). The patient recovered well after surgery.

The patient had gastroscopy and colonoscopy on Jan16, 2020, and the results showed hiatus hernia, gastric ulcerative lesion, chronic non-atrophic gastritis with erosion and tubular adenoma of the descending colon (0.4 * 0.5 cm). Despite the impact of COVID-19, the patient insisted on regular check-ups. The latest follow-up reexamination was at Jun 9, 2022, with normal gynecological B-ultrasound, gastroscopy and colonoscopy results. In addition, the patient's chest and abdominal CT and the cranial CT or MRI scans showed no abnormal nodule. Tumor markers including hCG, Carcinoembryonic antigen (CEA), Neuron-specific enolase (NSE), and Cytokeratin-19-fragment (CYFRA21-1) were within normal limits.

## Discussion and conclusions

GTN combined with primary malignant tumors in other organs is extremely rare in clinical practice. By reviewing English and Chinese literatures in PubMed, CHKD, WanFang, the National Library Reference and Consultation Alliance, using “gestational trophoblastic neoplasia”, “trophoblastic tumor”, “choriocarcinoma”, and “invasive hydatidiform mole” as key words. GTN combined with primary malignancies in other organs are rarely encountered, and to date, only 6 cases have been published in the literature. As for GTN combined with primary lung cancer, only 2 cases were published before [5, 6]. One case was a collision cancer in the lung consisting of a squamous-cell lung carcinoma and a metastatic choriocarcinoma [5]. The other was a mixed GTNs with synchronous primary lung cancer [6]. See Table 1.

**Table 1:** Reported cases of GTN with primary malignant tumors in other organs

Case	Year of publication	Nationality	First author	Age (years)	Clinical presentation	Menopause or not	Years after last pregnancy	Blood hCG (U/L)	GTN type	GTN site	Combined tumor(s)	Treatment	Outcome (as of follow- up)
1	1990	USA	Stetler-Stevenson [1]	32	Vaginal bleeding	No	1	5.8	PSTT	Uterus	Left immature ovarian teratoma and breast carcinoma	① The left salpingo-oophorectomy ② Total abdominal hysterectomy and right salpingo-oophorectomy ③ Bilateral mastectomies	–
2	1997	China	Liu [2]	25	A positive urine hCG test 40 days after curettage	No	–	–	Invasive hydatidiform mole	Uterus	Ovarian carcinosarcoma	① Chemotherapy ② Hysterectomy, bilateral Salpingo oophorectomy, omentectomy and appendectomy	–
3	2011	Turkey	Kara [3]	31	Enlargement of axillary lymph nodes	No	1.5	–	ETT	Cervix	Breast cancer	① Abdominal hysterectomy ② Pelvic radiation and chemotherapy ③ Mastectomy	–
4	2011	Korea	Kim [4]	44	Vaginal bleeding	–	–	85,400	–	Uterus	Thyroid carcinoma	① Chemotherapy ② Thyroid Surgery 3 months later	–
5	2015	China	Wang [5]	55	A previous history of choriocarcinoma with lung metastasis was admitted to department of Thoracic Surgery	–	–	16,500	Choriocarcinoma	–	Lung squamous cell carcinoma	① A right middlelobectomy with a partial resection of the superior lobe ② Chemotherapy	Survival, regular follow-up
6	2021	USA	Iyengar [6]	50	Heavy vaginal bleeding with the passage of clots	Indeterminacy	14	3704	Mixed GTNs (choriocarcinoma with minor components of PSTT and ETT)		Lung adenocarcinoma	① Laparoscopic total abdominalhysterectomy with bilateral salpingo-oophorectomy(TAH-BSO) and excision of pelvic lymph nodes ② Combination chemotherapy ③ Right thoracoscopy and wedge resection of two segments within the right lower lobe, as well as a singular segment in the right upper lobe	At present, she remains free of both cancers 2 years after her initial diagnosis

“–”: It was not mentioned in the literature

Based on vaginal bleeding and (or) metastatic features after hydatidiform mole suction, abortion, term delivery or ectopic pregnancy, GTN should be considered [7]. Postmolar GTN is usually diagnosed by hCG surveillance even without symptoms, such as case 2 and this case. GTN is accidentally found in some patients during the diagnosis of other diseases. Case 3 had a right axillary lymph node metastasis and was referred for Fluorodeoxyglucose positron emission tomography/computed tomography (FDG-PET/CT) scan. FDG-PET/CT revealed increased uptake on cervical region and biopsy of the lesion was consistent with ETT. Case 1, 4 and 6 had abnormal vaginal bleeding. About 60% of GTN are secondary to molar pregnancy. The level of hCG is the main basis to identify GTN, while the histopathology is not necessary. Follow-up hCG monitoring every 1–2 weeks is essential for early diagnosis of and management of postmolar GTN [7]. Imaging examination is an important means for pre-treatment evaluation. This case was diagnosed as GTN because the serum hCG rose again after evacuation of hydatidiform mole. In the imaging evaluation, lung cancer was found. Once the diagnosis of GTN is confirmed, CT of the chest and abdomen, chest X‑ray, MRI of the brain and pelvis should be performed. Pre-treatment imaging examination is important to find metastatic lesions or other primary neoplasms, help determine the clinical stage, prognostic score of GTN and make the therapy plan.

Once the diagnosis of GTN is confirmed, the clinical stage and prognostic score should be ascertained as soon as possible [8]. GTN should be staged and scored according to the International Federation of Gynecology and Obstetrics (FIGO) 2000 staging and prognostic scoring system [9]. The clinical stage of PSTT and ETT refer to the anatomical staging in FIGO, but the prognostic scoring system is not applicable [10]. In this case, the chest CT showed multiple small nodules in both lungs and mass shadows in the right lower lobe. The CT of head and abdomen showed no abnormalities. Lung biopsy confirmed primary cancer of the right lower lung, accompanied with lung metastasis of GTN, which is characterized by multiple, round, solid nodules in both lungs, mostly distributed under the pleura. Therefore, the preliminary diagnosis was GTN (stage III: 5 points) and lung cancer. GTN can be transferred to other sites, lung is the most common site of metastasis (80%), next come vagina (30%), pelvis (20%), liver (10%) and brain (10%). When the patient has symptoms of other organs or abnormal imaging findings, it is vital to determine whether the lesion is a metastatic or a primary lesion, which increases the difficulty of the staging and management of GTN.

Since there is more than one type of malignant tumor that originated from different systems, it should be emphasized to make comprehensive judgement, and choose a reasonable treatment plan according to the priorities of different tumors. This report presents a case of GTN that was further complicated by a synchronous primary lung adenocarcinoma. GTN may have uterine perforation and uncontrollable hemorrhage at any time, or liver and brain metastasis, which is life-threatening. Lung cancer may also have intrathoracic and extrathoracic metastasis. Fortunately, lung cancer has relatively mature targeted drugs. After confirming EGFR mutation by genetic testing, the thoracic doctors suggested to temporarily taking targeting therapy to control lung cancer while dealing with GTN.

GTN is highly sensitive to chemotherapy and has a relatively promising prognosis. The best regimen depends on stage and classification. Patients with a score ≤ 6 are considered as low-risk, and they should be treated with single-agent chemotherapy, either MTX or Act-D [11]. However, many scholars recommend hierarchical management for low-risk GTN [12–14]. Higher risk score of 5–6 and clinicopathologic diagnosis of choriocarcinoma are both associated with an increased risk of resistance. 2021 FIGO suggest that lowering the threshold for the use of multiple agent chemotherapy in these otherwise low-risk patients can be considered [7]. We took traditional combination chemotherapy using 5-FU and Act-D intravenously, and serum hCG titer dropped satisfactorily. Because of the high effectiveness of chemotherapy, hysterectomy is not highly recommended [7]. However, in selected patients, it is believed to help in reducing the cycles of chemotherapy, overcoming chemo-resistance, and treating acute haemorrhagic events [15]. Hysterectomy is not usually recommended at the beginning of treatment with high level of hCG, unless there is uterine perforation and/or uncontrollable bleeding [16]. This patient has no longer reproduction desire, the serum hCG significantly reduced and the uterine GTN lesion still existed after the second chemotherapy. In order to reduce the tumor burden, shorten the course of chemotherapy and reduce the side effects of chemotherapy, hysterectomy was performed during the third chemotherapy. We had full communication with the patient before the operation. Observing patients’ right is one of the most important components of providing humanistic and ethical care [17]. The serum hCG dropped to normal one month after the operation. After consolidation chemotherapy, the chest CT scan showed that the multiple small nodules in both lungs disappeared, so they were more inclined to lung metastasis of GTN. After 2 cycles of consolidation chemotherapy, we transferred her to thoracic department to receive lung cancer surgery. Thoracoscopic lower lobe of right lung lobectomy and the mediastinum lymph nodes removal were performed successfully.

During hysterectomy, a nodule was found accidentally at the end of sigmoid colon, which was completely removed and pathologically confirmed to be gastrointestinal stromal tumors (GISTs). GISTs are the most common mesenchymal tumors of the gastrointestinal tract. In adults, the stomach (60%) and small intestine (30%) are the most common sites of this tumor, whereas the duodenum (5%) and colorectum (< 5%) are rarely involved. Esophagus, appendix, mesentery, omentum, and retroperitoneum account for < 1% of the cases [18]. GISTs are neoplasms with a varying malignancy potential ranging from virtually indolent tumors to rapidly progressing cancers [19]. Surgery remains the gold standard for the management of primary disease. GISTs can be friable and surgery should be performed cautiously, avoiding violating the tumor pseudocapsule or causing tumor rupture [20]. In this case, gastrointestinal endoscopy was performed after complete resection of the tumor, and only tubular adenoma of the descending colon was found, which was completely removed with forceps.

Close follow-up after treatment is very important. The 2022 National Comprehensive Cancer Network (NCCN) Guidelines [10] pointed out that with respect to the follow-up for GTN patients after molar pregnancy, if the patient had normal HCG after treatment, no matter low-risk or high-risk GTN, imaging follow-up was not recommended because hCG is a reliable tumor marker for GTN patient. However, when the patient has other malignant tumors, regular imaging and other examinations are still needed to evaluate the efficacy and to determine whether there is relapse or not. This patient has been followed up till now, and is disease-free and has no signs of recurrence. In addition, patients with malignant tumors bear great mental and physical pressure. We can't ignore the spirituality and spiritual care while treating the disease. Spirituality and spiritual care play a crucial role in providing more effective treatment for patients [21].

Finally, FIGO suggests prophylactic administration of either MTX or Act-D chemotherapy at the time of or immediately following molar evacuation is associated with a reduction in the incidence of postmolar GTN to 3–8% [7]. So prophylactic chemotherapy with Act-D is administered in local hospitals. However, prophylactic chemotherapy at the time of uterine evacuation is controversial.

As far as we know, all cases reported in the literature are case reports. This paper reviewed the 6 cases have been published, and compared the clinical manifestations and management of different GTN combined tumors. We put more emphasis on the role of Multi-Disciplinary Treatment (MDT), and provide scientific, reasonable, standardized diagnosis and treatment strategies for patients to minimize misdiagnosis and mistreatment. The limitation of this study is that no statistical analysis was performed due to the very limited number of cases having been published.

GTN combined with primary malignant tumors in other organs is extremely rare in clinical practice and will increase the difficulty of GTN staging and treatment and bring more challenges to clinicians. When imaging examination reveals a mass in other organs, even if tumor metastasis is highly suspected, it should be considered the possibility of a second primary tumor. Clinicians should be more aware of GTN to reduce misdiagnosis and missed diagnosis. It should be emphasized to make comprehensive judgment, and choose a reasonable treatment plan according to the priorities of different tumors. We emphasize the importance of the collaboration of MDT and provide individualized treatment.

## Data Availability

All data generated or analysed during this study are included in this published article.

## Abbreviations

GTN: Gestational trophoblastic neoplasia
5-FU: 5-Fluorouracil
Act-D: Actinomycin-D
PSTT: Placental site trophoblastic tumor
ETT: Epithelioid trophoblastic tumor
β-hCG: Beta-human chorionic gonadotropin
MRI: Magnetic resonance imaging
D&C: Dilatation and curettage
CT: Computed tomography
FIGO: International Federation of Gynecology and Obstetrics
NCCN: National Comprehensive Cancer Network
FDG-PET/CT: Fluorodeoxyglucose positron emission tomography/computed tomography
GISTs: Gastrointestinal stromal tumors
